# Case report: The impact of percutaneous atrial septal defect closure in pulmonary hypertension with co-existing cor triatriatum sinister and multiple cardiac comorbidities

**DOI:** 10.3389/fcvm.2022.913391

**Published:** 2022-09-07

**Authors:** I-Hsin Tai, Tsung-Cheng Shyu, Kai-Sheng Hsieh, Ke-Wei Chen, Wan-Jane Tsai, Kuo-Yang Wang

**Affiliations:** ^1^Department of Medicine, College of Medicine, China Medical University, Taichung, Taiwan; ^2^Department of Pediatric Cardiology, China Medical University Children’s Hospital, China Medical University, Taichung, Taiwan; ^3^Pulmonary Hypertension and Pulmonary Vascular Disease Center, China Medical University, Taichung, Taiwan; ^4^Department of Cardiology, China Medical University Hospital, China Medical University, Taichung, Taiwan

**Keywords:** chronic heart failure, pulmonary hypertension, transcutaneous ASD closure, cor triatriatum, case report

## Abstract

Cor triatriatum sinister is a rare congenital anomaly characterized by the left-sided triatrial form of the heart. Diverse theories have been proposed regarding its formation, and the failure of incorporation of the common pulmonary vein into the left atrium (LA) during embryogenesis is the most widely accepted theory. Accordingly, cor triatriatum sinister may be associated with pulmonary venous obstruction and post-capillary pulmonary hypertension in the setting of restricted fenestration. A high proportion of patients with cor triatriatum sinister also have an associated secundum atrial septal defect. Pre-capillary pulmonary hypertension, which is unusual in patients with small atrial septal defects (<2 cm), is probably not as rare as some reports indicate, especially when combined with complex comorbidities. The conventional treatment strategy of atrial septal defect closure in patients with pulmonary hypertension, whether associated with cor triatriatum sinister or co-existing multiple cardiac anomalies, involves simultaneous repair with other cardiac surgical procedures. To the best of our knowledge, there is no reported clinical experience of percutaneous atrial septal defect closure in the literature. Herein, we present the case of an elderly female with pulmonary hypertension and coexisting cor triatriatum sinister, secundum atrial septal defect, and multiple cardiac anomalies. Despite optimal medical therapy, the biventricular failure deteriorated, and clinical stabilization could not be achieved. Transcutaneous atrial septal defect closure was then performed. Subsequent investigations showed an initial improvement (perhaps due to elimination of the left-to-right shunt) from this intervention, but the long-term impact did not appear favorable, likely due to multiple uncorrected cardiac anomalies. To the best of our knowledge, this is the first clinical report showing that partial treatment of combined pre- and post-capillary pulmonary hypertension by eliminating the pre-capillary component may have an initial benefit; thus, total surgical correction should be considered a definite therapeutic strategy unless contraindicated.

## Introduction

Pulmonary hypertension (PH) due to left heart disease (PH-LHD) caused by elevated left-sided filling pressures is the most common etiology of PH. PH-LHD is further classified into two subsets according to the presence of a pre-capillary component [combined pre- and post-capillary PH (Cpc-PH) or isolated post-capillary PH (Ipc- PH)] (1). Cpc-PH is considered a more serious subset than Ipc-PH (2). Previous research has pointed out that patients with PH-LHD have a worse clinical prognosis than those without PH-LHD (3), which may be because the diagnosis is delayed and there are no existing optimal treatments. The current ESC/ERS PH guidelines define PH as a mean pulmonary artery pressure (mPAP) of > 25 mmHg and define the two subsets of PH-LHD according to the diastolic pressure gradient (DPG) [the difference between diastolic PAP and pulmonary artery wedge pressure (PAWP)] and/or pulmonary vascular resistance (PVR). Previous studies have investigated whether Cpc-PH, as defined by current guidelines, predicts clinical outcomes, although the results have varied widely among patient groups (4). Categorizing two subsets of PH-LHD using DPG has been considered too restrictive (5); hence, comprehensive hemodynamic evaluation should always be performed for Cpc-PH diagnosis in case of clinical suspicion. Due to the absence of uniform consensus on the criteria for the diagnosis of Cpc-PH, effective treatment options have not yet been developed. Herein, we report a patient with Cpc-PH and co-existing multiple cardiac comorbidities partially treated by eliminating one of the suspected causes of Cpc-PH.

## Case presentation

**FIGURE 1:**
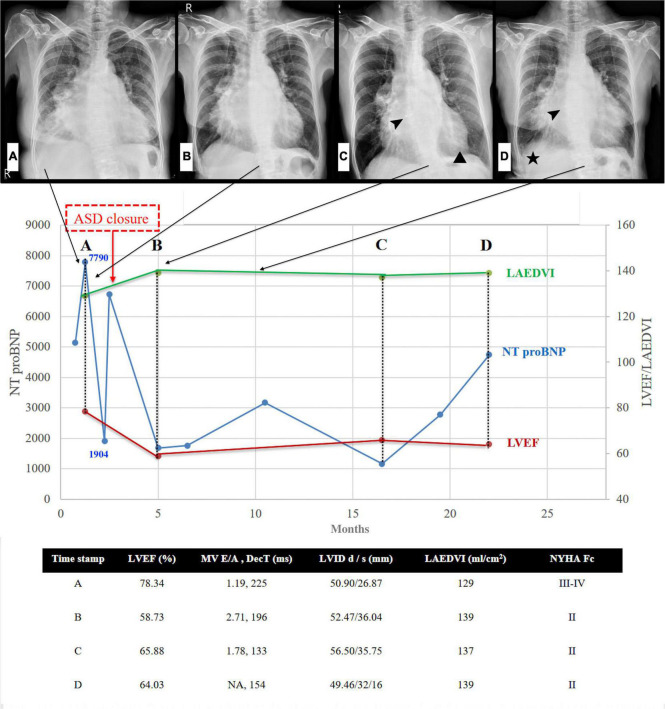
Left heart disease progression after ASD closure. Serial chest radiographs (arrows indicated the timeline of CXR) and heart failure markers (NT proBNP & Echocardiography) before and after ASD closure. **(A)** CXR: cardiomegaly, moderate right-side pleural effusion, and bilateral lung congestion **(B)** CXR: pleural effusion and lung congestion improved after diuretics **(C)** CXR: minimal pericardial effusion (triangle) 2.5 months after closure, arrowhead: ASD occluder **(D)** CXR: evident right pleural effusion (asterisk) with increased lung congestion 8 months after closure, arrowhead: ASD occluder. NT-pro BNP initial drop following ASD closure, gradually re-climbed 14-20 months later. Note that the climbing velocity is slower than that seen before ASD closure. In addition to the improving hyperdynamic LVEF and progressive enlarged LA volume, the other echocardiography parameter seems to have no significant change during the 20-month follow-up. ASD denotes atrial septal defect; CXR, chest X-ray; NT-pro BNP, N-terminal pro-brain natriuretic peptide; LVEF, left ventricular ejection fraction; LAEDVI, left atrial end-diastolic volume index; MV, mitral valve; DecT, deceleration time; LVID, left ventricular internal diameter; NYHA Fc, New York Heart Association Functional class.

**FIGURE 2:**
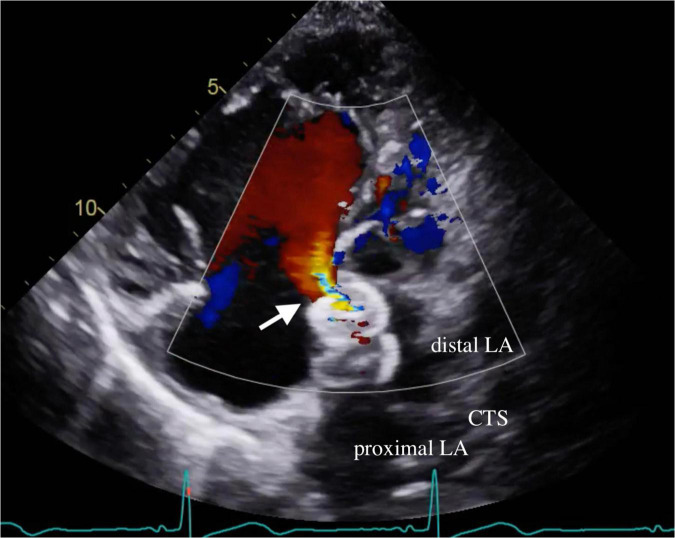
Residual ASD shunt. Transthoracic echocardiography demonstrated residual shunting (arrow) through the waist of the ASD device. ASD denotes atrial septal defect; CTS denotes cor triatriatum sinister; LA denotes left atrium.

A 62-year-old woman with long-term PH, valvular heart disease, atrial fibrillation (Af), and atrial flutter (AFL) on amiodarone, spironolactone, and valsartan/hydrochlorothiazide was admitted to the intensive care unit because of progressive dyspnea and bilateral lower limb edema for over 2 weeks. On physical examination, she was found to have bilateral basal rales, orthopnea, and grade IV bilateral lower-limb edema. The clinician-assessed New York Heart Association functional classification (NYHA class) was Grade III-IV. Chest radiography revealed an enlarged cardiac profile and lung congestion (Figure 1, Panel A). Electrocardiography revealed an AFL with biatrial enlargement. The N-terminal pro-brain natriuretic peptide (NT-pro BNP) level was 7,790 pg/ml on the day of admission. Transthoracic echocardiography revealed a hyperdynamic heart (ejection fraction >70%) with moderate aortic-mitral regurgitation and a large left atrium (LA). Additionally, the LA was bisected by a membranous structure into two distinct chambers (proximal and distal chambers) without a transmembrane pressure gradient on color Doppler, which suggested a non-obstructive cor triatriatum sinister (CTS) and secundum atrial septal defect (ASD) underneath. Subsequent three-dimensional transesophageal echocardiography confirmed the presence of a 1.5 cm secundum ASD in the distal LA chamber. Nuclear imaging and computed tomographic pulmonary angiography excluded the possibility of chronic thromboembolic disease. After heart failure was controlled with intravenous diuretic medication, cardiomegaly improved (Figure 1, Panel B), NT-pro BNP levels decreased (Figure 1, 7,790 to 1,904 pg/mL), and lower limb edema reduced. Hemodynamic evaluation with right heart catheterization (RHC, Table 1) showed moderate PH, probably because of the prevalent systemic-to-pulmonary shunt, without a significant pressure gradient between the proximal and distal chambers of the LA. This improvement in the clinical condition after intensive care with diuretic agents, however, was not long-lasting. After 1 week of follow-up, NT-pro BNP levels had increased to pre-treatment levels (1,904–67,25 pg/mL). Total surgical structural correction (CTS resection, ASD closure, and mitral and aortic valvular repair) was planned; however, the patient declined to undergo this procedure. The patient eventually underwent transcutaneous ASD closure using an 18-mm Amplatzer septal occluder, following which trivial residual interatrial shunting was detected on color Doppler echocardiography (Figure 2). The patient’s condition improved significantly for several months after transcutaneous closure of the ASD. However, with time, the heart failure worsened (Figure 1, NT proBNP).

**TABLE 1:** Right heart catheterization after heart failure control.

PAP (s/d/m), mmHg	69/20/36
PAWP (s/d/m), mmHg	15/15/12
LVP (s/d/m), mmHg	152/12/21
RAP (s/d/m), mmHg	13/10/7
LAP (s/d/m), mmHg	16/13/11
PVR, WU	4.06
TPG, mmHg	24
DPG, mmHg	8
Qp, L/min	5.91
Qs, L/min	2.74
Qp:Qs	2.16

## Discussion and conclusions

### Hemodynamic evaluation and correlation in the presence of multiple cardiac comorbidities

The RHC data ostensibly fulfilled the criteria for pre-capillary PH (6). However, when interpreting these values, we need to take into consideration that the patient’s volume status may have influenced the pulmonary hemodynamics and the subsequent measurements. As the patient had undergone diuretic treatment before right heart catheterization, the measured pulmonary arterial wedge pressure (PAWP) may have been erroneously reduced to < 15 mmHg (1, 7). Left ventricular end-diastolic pressure (LVEDP), a more reliable surrogate for left atrial pressure, was 21 mmHg; however, in the presence of mitral and aortic valve disease, PAWP and LVEDP may not be assumed to be interchangeable (7, 8). Furthermore, the patient’s diastolic pressure gradient (DPG) and transpulmonary pressure gradient (TPG) were 8 and 24 mmHg, respectively, which implied the combined presence of passive and reactive PH (1, 3, 9), making Cpc-PH the most likely assessment.

### Role of atrial septal defect closure

The key indication to close ASD in PH is the value of pulmonary vascular resistance (PVR) or pulmonary vascular resistance index (PVRI) (1). The ESC recommendations (1) for the correction of prevalent systemic-to-pulmonary shunts in PH are a PVR < 2.3 Wood units (WU) (PVRI < 4 WU.m^2^) for it to be correctable, > 4.6 WU (PVRI > 8 WU.m^2^) for it to be not correctable, and between 2.3 and 4.6 WU (PVRI 4–8 WU.m^2^) to require individualized evaluation, which was the case in our patient. In patients with a 1.5-cm isolated ASD, who are likely to be asymptomatic, the magnitude of the left-to-right shunt with multiple structural anomalies and arrhythmia remains unclear, for it is unknown whether the left-sided filling pressure is also increased (10). Recently, a prospective study used baseline PAPm to predict whether PAPm could further decrease after ASD closure in 209 patients with PH (11). The optimal cutoff value of baseline PAPm without PAH-specific medication was 35 mmHg (the area under the curve was 0.919, *p* < 0.001), which is nearly equal to that of our patient (PAPm:36 mmHg). ASD closure may block the left-to-right shunt, but the long-term effect of the absence of decompression for irreversible PH (12) warrants future research.

### Strengths and limitations of management

Transcutaneous closure has significant benefits in ASD patients with PH owing to its minimal invasiveness and low complication rates. However, if a patient is undergoing other cardiac surgeries, the surgeries are typically performed together. CTS is a rare congenital cardiac anomaly in which a fenestrated fibromuscular membrane subdivides the LA into two chambers. When the fenestration is small, the obstruction mimics mitral stenosis, which may further aggravate Af (13) and increase the risk of stroke (14). Surgical resection of the CTS membrane seems curative; however, our experience indicates that transcutaneous balloon dilatation can also achieve clinical improvement (15). Given that Doppler color flow mapping with RHC data showed no transmembrane pressure gradient in our patient, surgical resection was probably not the ideal choice. According to the American Heart Association guidelines on valvular heart disease, valve replacement is a reasonable approach in patients with moderate AR or MR undergoing other cardiac surgeries (16).

The serial quantitative echocardiography demonstrated normalized hyperdynamic left ventricular ejection fraction (from 78.34 to 58.73%) and functional class. Concerning the absence of sepsis or any other form of non-traumatic shock, ASD-related significant systemic pulmonary shunting might be a reasonable explanation for hyperdynamic left ventricular ejection fraction. The severity of aortic regurgitation represented by the left ventricular volume seemed unchanged compared with that before ASD closure. Notably, the left atrial volume increased one month following percutaneous ASD closure. With multifactor such as poorly controlled atrial fibrillation/flutter or perhaps worsened mitral regurgitation, making it hard to differentiate from effect after ASD closure. It seems that the rate of worsening heart failure, as represented by NT-pro BNP (Figure 1), is slower after ASD closure than before. The long-term outcome of intra-atrial shunt blockade in our patients with combined pre- and post-capillary PH remains unknown, and to the best of our knowledge, no relevant investigations have been conducted. However, the current evidence of persistent PH with ongoing worsening of LV function suggests that partial repair of Cpc-PH may be achieved by eliminating the pre-capillary component in a short period of palliation. This suggests that total correction of structural anomalies should be considered in the management planning of Cpc-PH due to the complex hemodynamic situation.

## Data availability statement

The original contributions presented in this study are included in the article/supplementary material, further inquiries can be directed to the corresponding author.

## Ethics statement

Written informed consent was obtained from the participant/s for the publication of this case report. Written informed consent was obtained from the individual(s) for the publication of any potentially identifiable images or data included in this article.

## Author contributions

W-JT and K-SH collected the clinical data and help complete the manuscript. I-HT analyzed and interpreted the patient data regarding the right heart catheterization and was a major contributor to writing the manuscript. T-CS and K-WC performed the diagnostic and therapeutic intervention procedure and made critical revisions to the manuscript. K-YW designed the research and made critical revisions to the manuscript. All authors read and approved the final manuscript.

## Abbreviations

Af: Atrial fibrillation
AFL: atrial flutter
Cpc-PH: combined pre- and post-capillary PH
DPG: diastolic pressure gradient
Ipc- PH: isolated post-capillary PH
LA: large left atrium
LVEDP: left ventricular end-diastolic pressure
mPAP: mean pulmonary artery pressure
NT-pro BNP: N-terminal pro-brain natriuretic peptide
NYHA class: New York Heart Association functional classification
PAWP: pulmonary artery wedge pressure
PH: Pulmonary hypertension
PH-LHD: Pulmonary hypertension due to left heart disease
PVR: pulmonary vascular resistance
RHC: right heart catheterization
ASD: secundum atrial septal defect
CTS: triatriatum sinister.
